# Assessment of scalability and performance of the record linkage tool E-PIX^®^ in managing multi-million patients in research projects at a large university hospital in Germany

**DOI:** 10.1186/s12967-020-02257-4

**Published:** 2020-02-17

**Authors:** Christopher Hampf, Lars Geidel, Norman Zerbe, Martin Bialke, Dana Stahl, Arne Blumentritt, Thomas Bahls, Peter Hufnagl, Wolfgang Hoffmann

**Affiliations:** 1 Institute for Community Medicine, Section Epidemiology of Health Care and Community Health, University Medicine Greifswald, Ellernholzstr. 1-2, 17475 Greifswald, Germany; 2 Independent Trusted Third Party, University Medicine Greifswald, Ellernholzstr. 1-2, 17475 Greifswald, Germany; 3 Charité – Universitätsmedizin Berlin, Charitéplatz 1, 10117 Berlin, Germany

**Keywords:** Record linkage, Duplicate detection, Data quality, Patient data, Identity management, Data privacy protection

## Abstract

**Background:**

The identity management is a central component in medical research. Patients are recruited from various sites, which requires an error tolerant record linkage method, to ensure that patients are registered only once. In large research projects or institutions, the identity management has to deal with several thousands or millions of patients. In environments with large numbers of patients the register process could lead to high runtimes caused by record linkage. The Central Biomaterial Bank of the Charité (ZeBanC) searched for an identity management solution, which can handle millions of patients in large research projects with an acceptable performance. The goal of this paper was to simulate the registration of several million patients using the E-PIX service at Charité – Universitätsmedizin Berlin. The E-PIX service was evaluated in terms of needed runtimes, memory requirements, and processor utilization. A total of at least 20 million patients had to be registered. The runtimes to register patients into databases with various sizes should be examined, and the maximum number of patients, which the E-PIX service could handle, should be determined.

**Methods:**

Tools were set up or developed to measure the needed runtimes, the memory used and the processor usage to register patients into various sizes of databases. To generate runtimes close to reality, modified patient data based on transposed real patient data were used for the simulation. The transposed patient data were sent to E-PIX to measure the runtimes of the registration process. This measurement was repeated for various database sizes.

**Results:**

E-PIX is suitable to manage multi-million patients within a dataset. With the given hardware, it was possible to register a total of more than 30 million patients. It was possible to register more than 16 thousand patients per day into this database.

**Conclusions:**

The E-PIX tool fulfills the requirements of the Charité to be used for large research projects. The use of E-PIX is intended for the research context in the Charité.

## Background

The Central Biomaterial Bank of the Charité (ZeBanC) intends to externalize an existing identity management. For this purpose, the requirements of the ZeBanC were defined and a suitable solution to fulfill these requirements was searched for. One requirement was that several million patients need to be registered and managed. It should be possible to manage at least 20 million patients with the identity management system. In addition, it should still be possible to register large datasets in parallel to the regular daily workload, for example the participants of a new study, even when several million patients are registered in the database. Independent of the number of patients it should be possible to register at least several thousand patients per day. In a first examination in 2015 the Mainzelliste [1], a solution of the Institute of Medical Biostatistics, Epidemiology and Informatics in Mainz was evaluated (version 1.4.2). This version was found not to be suitable for productive use in ZeBanC, mainly due to poor performance. Thus, another solution was needed. A potential candidate was identified: the Enterprise Identifier Cross-Referencing (E-PIX) of the Institute for Community Medicine of the University Medicine Greifswald [2]. This publication describes the performance of the E-PIX in terms of the previously defined requirements.

The linking of data from medical care and research involves ethical, legal, data privacy and data protection requirements that need to be covered adequately. In the context of clinical care, identifying information and medical data are typically collected simultaneously. These data are intended for medical care, thus a use in research is a change from the original purpose and constitutes a so-called secondary use [3]. Use of anonymized patient care data is permitted, but results in the permanent loss of the relation to the individual patient [3]. A pseudonymized relation between identity and medical data can be a solution, however, this requires an informed consent from each participating patient. A pseudonym links the corresponding personally identifying information (PII) with the patient’s medical data. A pseudonym must be a unique identifier for each particular patient. This pseudonymization is usually administrated in an identity management system, which generates at least one unique pseudonym for that patient and assigns this to the PII. This pseudonym can then be used in the research context, and assures that all identifying information of a patient will be concealed. At the same time, the usage of a pseudonym enable a re-identification of a patient, which is a prerequisite for re-contacting a patient, that is desired in particular cases. For example, many studies adopt a policy to inform a participating patient in case of relevant findings [3]. In addition, one strategy to find suitable participants for a new study is to match patient data of existing studies to the inclusion criteria of the new study [3]. Then, a re-contact is required to invite this possible participant into the new study.

Technically, patients are registered into the identity management or, respectively, into a patient list, which is part of the identity management and serves for storing PII. The term registration means that the PII are transferred to the identity management. These data are (a) checked for whether the corresponding patient is already registered, and (b) if he or she is not registered, to store them in the patient list. If the patient has not already been registered before, a new unique pseudonym is generated and the patient is registered within the identity management together with this pseudonym.

To check whether the patient has already been registered, in order to prevent duplicates, a record linkage is necessary. Record linkage means that the PII of a patient will be compared with the PII of all of the already registered patients. In an ideal case, two PII are exactly the same and it is obvious that the corresponding patient has already been registered before and the current case is a duplicate to the previous entry.

In practice however, PII are often collected at different sites and it is possible that some of these data are incorrect or incomplete. Whenever data are manually entered, typing errors, e.g. transposed (a) numbers in dates, (b) letters in names or (c) attributes could be inserted accidentally. Thus, the record linkage has to tolerate faulty data and at the same time be able to detect those mistakes. Otherwise, duplicates or incorrect relations may be created. To avoid a duplicate, two steps are performed. Firstly, if two compared PII are very similar or identical, it is assumed that they belong to the same person. This is a so-called match. Secondly, the PII are assigned to one already registered patient, which is a so-called merge. If the PII of one patient is erroneously not matched to one already registered PII of the same patient, but rather is considered a new patient in the patient list, this is called a synonym error [4, 5]. This error is often reversible with additional data [4]. If on the other hand the PII of several patients are erroneously merged to one patient in the patient list, the result is a homonym error [4, 5]. This error is usually irreversible or requires considerable efforts to detect and reverse [4]. At best, these errors can be avoided initially by high quality data entry. If they occur anyway, the identity management system needs to support the detection of these errors and should be able to prepare them for manual fixation.

The E-PIX was developed by the Institute for Community Medicine of University Medicine Greifswald as part of the GANI_MED study (2009–2014) [6]. It was first published within the DFG funded MOSAIC project (2012–2015) [2], under GNU Affero General Public License v3 [7] and, thereby, it is open source. E-PIX supports the detection of synonym errors by an error tolerant record linkage, and supports their resolution.

E-PIX is a first-layer pseudonymization service, which represents the patient list. Following the concept of a Master Patient Index (MPI) [4, 8], it generates a unique identifier called MPI ID for every patient. This identifier serves as a pseudonym to link the corresponding PII and conceal the identity. The PII are not used to directly link medical data in the research context. For this purpose the pseudonym will be used exclusively.

E-PIX implements an identity concept which distinguishes between primary and secondary identities [8]. Multiple collections of PII in various sites, e.g. different hospital departments, could lead to different versions of PII, caused for example by a typing error in one of the sites. Every registration initiates a record linkage process. E-PIX detects non-identical versions of PII by an error tolerant record linkage. The corresponding PII are linked together. Each PII version now represents an identity. One of the identities of the same patient is deemed the primary identity, which is, to the best of our knowledge, free of errors. Each slightly deviant identity becomes a secondary identity and is used to link these PII to the correct patient. In case of recruitment at various sites, for example, the non-identical PII is used as an additional matching option for record linkage. In case of a request to E-PIX, the correct patient is additionally linked with the non-identical PII. Another request to E-PIX returns the primary identity, so that the requesting site could store or update the correct PII from E-PIX.

The record linkage process determines whether a given new PII has already been registered in E-PIX previously. This means that each already registered identity is compared with the new PII. E-PIX distinguishes between four possible results of record linkage:

- Perfect Match: all PII are exactly the same with one registered identity, thus the new set is an unambiguous duplicate
- Match: there are small differences between the PII and at least one already registered identity, but the similarity is high enough to automatically link it to the same person
- Possible Match: the PII has a certain similarity with at least one already registered identity, but the similarity is not high enough for an automatic linking and needs manual intervention
- Non-Match: the PII and all registered identities have large differences, so that the new PII really belongs to a new person.

For example, a common family name in Germany is Meier. This name exists in various variants like Maier or Meyer. If Maier is entered instead of Meier, then the two PII of a patient are not the same. E-PIX will detect this. This situation is not a Perfect Match. However, it is still a Match, if all other attributes are identical or very similar. In this case the PII with both surname variants are related to the same person.

To determine the matching type, E-PIX uses the probabilistic method suggested by Fellegi–Sunter [9]. Each attribute, which is configured as a part of record linkage, is compared using an appropriate comparison algorithm. The most used algorithm in E-PIX is Levenshtein [10], but there are other methods implemented, e.g. the Cologne phonetics [11]. Users can implement their own algorithms, which can be used for comparing the attributes. In addition, the threshold and the weight of each attribute can be configured separately.

For better attribute comparison, a preprocessing is implemented. This specifies the standardization of an attribute, before it is compared with the existing dataset. For example, all lower case letters could be replaced with the corresponding upper case letters, to prevent differences caused by case-sensitivity.

In a large database, the record linkage is a time consuming process. To reduce the runtimes for duplicate detection, E-PIX has a so-called blocking mechanism implemented. This is a fast but less accurate method to determine similarities. For this purpose, a small subset of attributes (for example, first name and birthdate) of two records were compared with lower thresholds. If there is sufficient similarity, the records were compared again using a larger subset of attributes (for example first name, last name, gender and birthdate), thus achieving higher accuracy by higher thresholds. If there is not sufficient similarity, the records are classified as non-match.

The E-PIX provides a web-based graphical user interface and an application programming interface, which is requested in SOAP format.

The main goal was the simulation of the use of E-PIX in version 2.8.2 to evaluate the performance of managing several million patients. For this purpose, suitable modified real patient data were generated and registered into the E-PIX service. The E-PIX was evaluated in terms of needed runtimes, used memory and utilized processor capacity. The focus is on patient registration, because this process, especially the record linkage part, could produce high runtimes. This was observed in other identity management systems and in previous versions of E-PIX. In total, at least 20 million patients should be registered into one E-PIX instance. The goal of the evaluation was to find limits regarding the maximum number of registered patients and the maximum number of patients that could be registered per day into a dataset already containing several million patients. Furthermore, the runtime for one patient registration into databases of various sizes should be measured. Based on the obtained results, it was estimated how E-PIX would perform in a production environment.

## Methods

### Preparation of simulation data

For simulating a registration, we used random attributes with arbitrary values. The only condition was that the values were valid for the corresponding attributes. For example, a date value must not include letters. In this case, the E-PIX would detect this error and abort the process of registering and return an error message, which describes this mistake. However, random data could lead to different runtimes than real data. Thus, for this simulation, the used data should be as real as possible.

For the benchmark, we only used PII and no medical data. The basis was a real patient dataset with 87,112 patients without missing values from the Pathology of the Charité. These data could include faults like typing errors. The identifying attributes were randomly mixed for anonymization. Therefore, there was an irreversible loss of any personal reference. The anonymized patient data were used to permute datasets up to 60 million patients by swapping randomly the individual attributes with consideration of the original frequency distribution of the attributes. For this purpose, the selected attributes were first name, surname, gender, birthdate and residential address. The address contains street, house number and city of residence. The postal code was added, because this information was not included in the original data. The resulting addresses were not real, but that was not necessary, because the address was not used for a comparison in the record linkage process. The resulting dataset has the same distributions of all relevant attributes as the original data. For example, some surnames are more common than others. The simulation dataset contains mainly German first names, surnames and addresses. Not all of the given attributes were used for the record linkage. Especially for blocking, only the first name and birthdate were used. Assuming that after the blocking the similarity of the attributes first name and birthdate is high enough, the attributes first name, surname, gender and birthdate were used for record linkage. It is possible to configure other attributes and thresholds for the blocking and record linkage. However, for the simulation the default configuration was used.

Additionally, under real world conditions, it may happen that one or more attributes are missing. For benchmarking the E-PIX service, the datasets for registration were always complete. In terms of runtimes, this is the most expensive use case, because the maximum amount of data has to be transferred and compared. However, it is the best case for data quality and completeness, when all or most attributes are set. Nevertheless, E-PIX is able to detect duplicates with incomplete PII, given that the required fields in the respective configuration are non-missing.

### Preparation of evaluation

The benchmarks and all tests were performed on a dedicated computer system. Table 1 shows the specification of the system. Hence, the measurements were not influenced by changes in hardware or software.

**Table 1:** Specification of the computer system used to carry out the benchmark tests

Component	Description
Processor	Two processors, each with 8 cores and 16 threads (Hyper-threading) and a clock rate of 2.9 GHz (boost up to 3.8 GHz)
Memory	128 GiB RAM, but the E-PIX service, respectively the Java Virtual Machine (JVM) has a limit of 120 GiB. The remaining eight GiB were reserved for other processes like the database and the client, which send requests to the E-PIX service
Operating System	Microsoft Windows Server 2012 R2 in 64 bit variant

Three indicators were measured: (a) the runtimes, (b) the used memory and (c) the processor utilization. The runtime indicates the duration of registering one or several patients; the used memory shows the needed size of memory in various situations and various numbers of registered patients; the processor utilization displays whether the workload is split to all cores and threads, respectively, and whether the processor is running at maximum capacity. The only task of the computer system was to run the E-PIX service and the necessary applications for this purpose e.g. the database, the application server, and the client for sending requests. This ensures that other processes did not influence the measured runtimes, the used memory, or the processor utilization. To measure these values, a client was developed, which requested the E-PIX service. E-PIX provides a SOAP interface, which offers various methods to register patients on the basis of PII, query PII, complement or update existing PII and resolve or query possible matches. The client converts the PII into the SOAP format to transfer these data to the E-PIX service. All measurements were based on the registration with the SOAP interface of E-PIX. The client sends requests to the E-PIX service, for example to register a patient and wait for the response. The client was running on the same computer system as the E-PIX service. Hence, network latencies were not measured. E-PIX is open source, so it would have been possible to measure the runtimes of particular separate components. However, this information was not our area of focus, because the runtimes should be collected for a system in productive use requesting the full E-PIX service.

### Runtimes

**Fig. 1:**
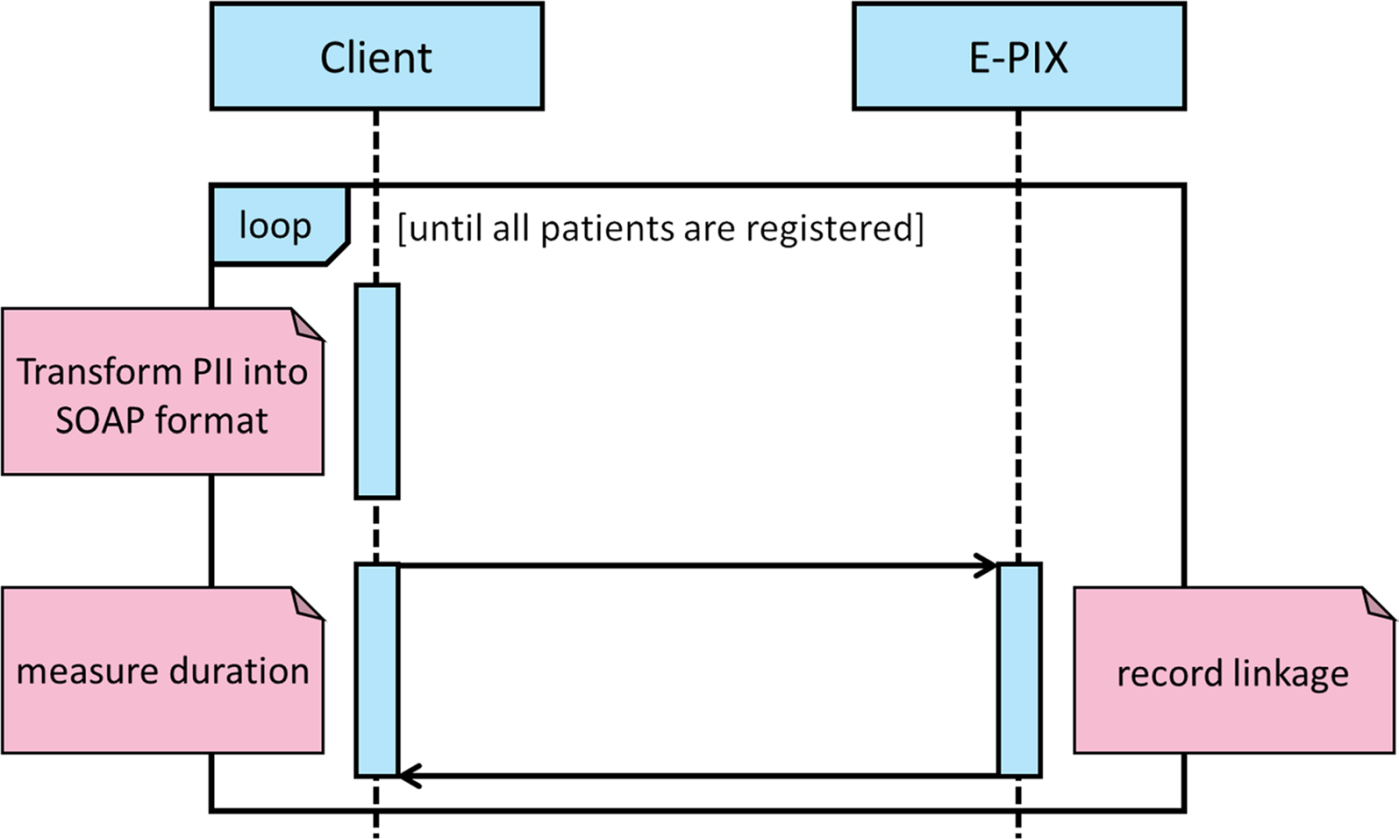
Communication between client and E-PIX. The client transforms the PII into the SOAP format, sends this data for registration to E-PIX and begins the runtime measurement. After registration, the client receives the MPI from E-PIX. The runtime measurement is completed and a new registration can be started

The developed client measured the runtimes in two ways. On the one hand, the runtimes were measured sequentially. That means the PII was transformed into the SOAP format and transferred to the E-PIX service. The time between the completed process of sending the request and the receipt of the completed response is the runtime to register one patient. After that process, the next PII will be registered. Thus, each registration is independent from other registrations and is not influenced by those in terms of runtime. Figure 1 shows the communication between the client and E-PIX.

Alternatively, runtimes were measured for parallel registrations. This could be done in two forms. First, several PII will be transmitted within one request to the E-PIX service. There is only one response, hence the runtime of one PII registration is part of the total time of all registrations within one request. Second, several requests with one or multiple PII were sent parallel to the E-PIX service. In this case, the requests influence each other. This is the typical case in a productive system, when several systems communicate parallel with the identity management.

### Memory

The memory usage is measured for several reasons: (a) to determine how much memory is needed to operate the E-PIX service with a particular amount of registered patients, (b) to analyze the behavior in the productive use when PII were registered and (c) whether or not the needed memory differs in certain situations from the typical values. For the measurements several million patients were registered. It was not necessary to measure the memory usage after every single registration. Hence, after every 500th registration the memory usage was recorded. This was considered sufficient to show a trend. The memory usage is recorded for every process, so it is possible to check whether certain processes limit the memory for E-PIX. The recorded used memory for E-PIX includes the application server, because E-PIX is only a servlet and needs an application server to operate. In one benchmark, the application server was restarted after 500,000 registrations. After the application server was restarted, memory usage was recorded again to determine whether memory use is comparable with the use before a restart.

### Processor

Several key figures are of interest to assess processor utilization. In addition to the pure processor load, the ability of multithreading is important. To check whether other processes, besides the application server and E-PIX using the Java Virtual Machine (JVM) and the necessary database, are using the processor, all processes were recorded. When another process heavily used the processor, the runtimes of E-PIX can be influenced. To record the processor load, the Windows Performance Toolkit [12], respectively the Windows Performance Recorder (WPR) was used. WPR implements the Event Tracing for Windows (ETW), which has very little impact on the performance [12], so it is not expected that this tool heavily influenced the runtimes.

### Benchmarks

The first and second benchmark should analyze the behavior of the runtimes. (a) First, 3 million patient data were registered sequentially. At the beginning, the database was empty. After the registration of 3 million patient data, the application server was restarted. Used memory and the runtimes of following registrations were measured. To analyze the influence of restarts on the runtimes, (b) a registration process with cyclic restarts was performed. For the second benchmark, the database was cleared and 6.5 million patient data were registered. After a registration cycle of 500,000 patients, the application server was restarted (in total 12 times). In productive use, cyclic restarts are not practical, but in this case the influence of restarts on runtimes and memory should be determined. (c) To simulate and determine the runtimes of larger databases with millions of patients, the data were not registered conventionally, but the database of E-PIX was filled directly with the patient data. This is only possible with modified permutated data, because this data were generated without duplicates. After this process, 100,000 new patient records were entered into E-PIX and the corresponding values were measured. After registering those 100,000 new patient records, the database was cleared and the previous pre-filled patients were increased by 5 million patient data. Thus, the first database contained 10 million patient data, the second 15 million and so on until 35 million were reached. This method was used to determine the maximum number of patients, which can be handled by E-PIX with the given computer system.

## Results

### Runtimes

**Fig. 2:**
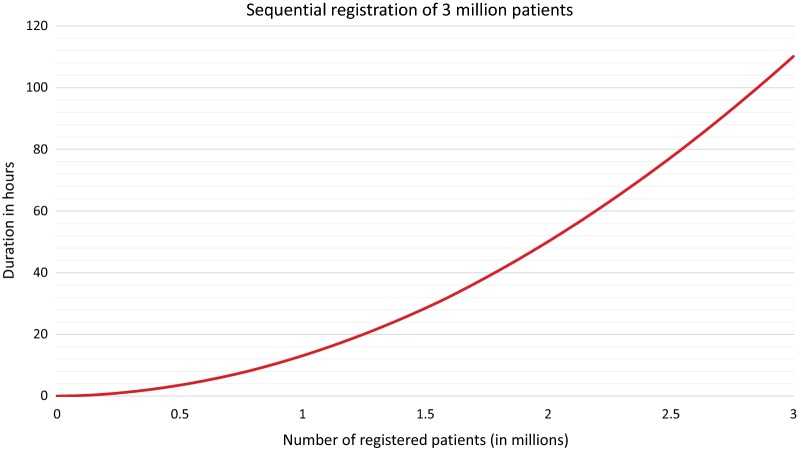
Sequential registration of 3 million patients. Duration for registration of up to 3 million patients into E-PIX of version 2.8.2. The requests were sent sequentially with the system running continuously without restarts

After starting the application server, the 3 million registrations of the first benchmark were conducted sequentially. The entire registration process needed 110 h, respectively 4 days and 14 h. It was found that there is a correlation between duration and number of registered patient data. Increasing the number of patient data registered led to an increase in runtime. This was expected, because a higher number of registered patients results in a higher number of comparisons in both the blocking process and record linkage. The average runtime per registration was within the first 100,000 registrations 6 ms and after 2.9 million registrations 252 ms. The trend of runtimes in this range was approximately linear, but the underlying mathematical process is a decision algorithm, which has a polynomial time term. Figure 2 shows the duration for registering the 3 million patient data.

**Fig. 3:**
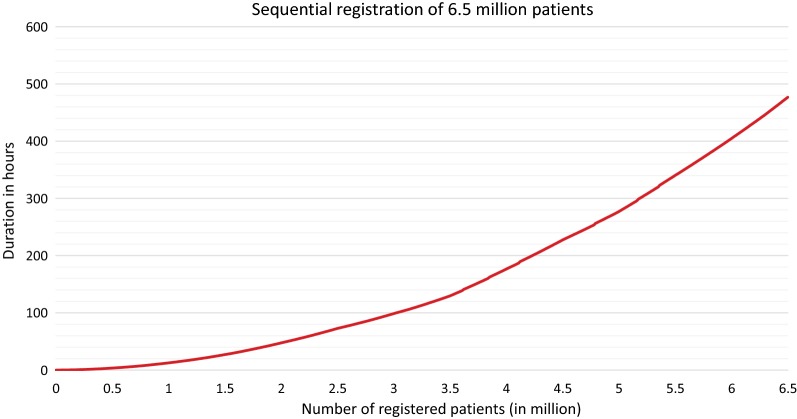
Sequential registration of 6.5 million patients. Duration for registration of up to 6.5 million patients into E-PIX of version 2.8.2. The requests were sent sequentially with the system being restarted after every 500,000 registrations. After some restarts, the runtimes were lower than expected

Within the second benchmark of registering 6.5 million patients, after several restarts, the runtimes were reduced slightly. At the first 3 million registrations, in comparison with the 3 million patient registrations in the previous section, it could be determined that one entire registration cycle has significantly reduced runtimes. In the following 3.5 million registrations, respectively seven registration cycles, compared to the extrapolated expected runtimes based on the data of the faster registration cycles, reduced runtimes was monitored another two times. In case of reduced runtimes, per 100,000 registrations, this process is between 46 and 59 min faster. This behavior deviates from the expectations, but the service still worked correctly. This means that the patients were still registered. This was ensured by checking the number of patients entered into the database. It is assumed that this unexpected behavior is in connection with the restarts. Another possible reason could be the different size of patient data. But, the average size of patient datasets over several thousand datasets is very similar. Other measurement variables like the used memory and the processor utilization were not influenced. Figure 3 shows the runtimes of 6.5 million registrations. The entire registration process of 6.5 million patients needed 469 h, respectively 19 days and 13 h. In comparison, the registration of 3 million patients as shown in the first benchmark without restarts needed 110 h, with restarts only 99 h. Although the restart process is not included, the process of five restarts is less time-consuming than the difference of 11 h. Hence, the runtimes of the first 500,000 registrations of both runs were very similar.

**Fig. 4:**
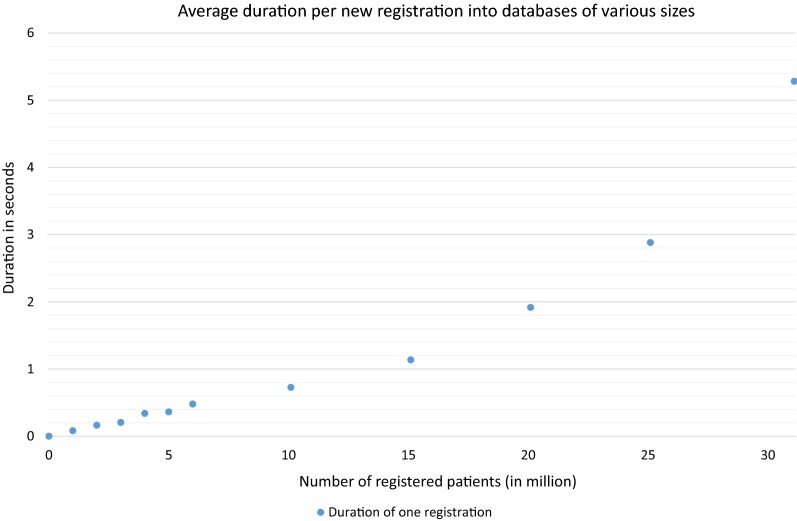
Average duration per new registration into databases of various sizes. Average duration to register one new patient into a database of E-PIX version 2.8.2 with a certain number of pre-registered patients. The shown runtimes represent on the average of 100,000 registrations

Figure 4 shows the average duration of a patient registration of the third benchmark. It includes the runtimes of up to 6.5 million registrations of benchmark two plus the runtimes up to 30 million patients directly transferred into the database. The registration of one new patient into a database of 10 million patients needed an average about 727 ms, into a database of 20 million patients needed 1.92 s and into a database of 30 million patients needed 5.28 s. Accordingly, the registration of 100,000 patients into a 10 million database needed 20 h and 11 min. Within a database of 20 million already registered patients, 53 h and 13 min, respectively 2 days, 5 h and 13 min were needed. For the registration into a database of 30 million patients, 146 h and 41 min, respectively 6 days, 2 h and 41 min were needed. Based on these data, it was determined how many patients can be registered in 1 day into existing databases of different sizes. Table 2 shows the number of patients which could be registered per day in corresponding databases of sizes between 0 and 30 million pre-registered patients. It shows that it is possible to register several thousand patients within a single day, even if the database already contains up to 30 million patients. This performance allows for the registration of new large cohorts.

**Table 2:** Number of patients who can be registered in 1 day, depending on various sizes of the existing database

Registered patients	Registrations per day
0	1,460,594
1 million	766,026
2 million	496,069
3 million	375,129
4 million	244,452
5 million	190,867
6 million	178,675
10 million	118,845
15 million	76,056
20 million	45,094
25 million	29,990
30 million	16,361

The behavior shows reciprocal proportionality as expected. The patients were registered sequentially. In the third benchmark, only 100,000 patients were registered during one cycle in this test. Consequently, the duration of the registration process into a database of 10 million prefilled records was less than a day and, thus, the number of patients was extrapolated. All other numbers are based on the real number of patient registrations with cyclic restarts

### Memory

In the first benchmark, the memory usage of the JVM was at the initial start 1.03 GiB. Within the first 254,000 registrations, the memory usage increased up to 7.11 GiB and over the next 55,000 registrations rapidly decreased down to 0.98 GiB. This behavior could be reproduced, also based on other database sizes with corresponding memory usages. After about 309,000 registrations, the memory usage correlated with the number of registered patients in an approximately linear fashion. In rare cases, the memory usage decreases by a few or many GiB. It is assumed that in those moments the garbage collector freed the memory of the JVM. The highest memory usage within the registration of 3 million patients was 21.44 GiB after around 2,800,000 registrations, but after the last registration it was only 13.62 GiB. The registered patients were cached in memory, thus the more patients were registered, the more memory is needed, even when no register process is running.

In the second benchmark, the memory usage was lower immediately after a restart than without a restart. However, it could be observed that the memory usage rapidly increased, to even higher demands than compared to the benchmark without restarts. In case of a necessary restart of the service in practical use, this memory consumption should be considered of the service with default configurations.

In the third benchmark, the application server could not be started with a database of 35 million patients. This is due to the required memory, which was not available in the given computer system. The initial memory usage after restart of the application server are shown in Table 3. At the beginning of one cycle, the allocated memory increased rapidly within the registration process. This was expected, because for example caches had to be created. With a database of 20 million already registered patients at the start, the limit of 120 GiB was reached. However, the service still worked correctly. With an initial database of 30 million patients, the behavior of memory usage changed, so the memory usage decreased and stayed nearly constant during the further registration process. This behavior was unexpected, but we could reproduced it in several re-runs. In any case, the patient data were still registered correctly according to the total number of registered records. Finally, with an initial database of 35 million patients, the needed memory for loading all patient data at the start of the application server exceeded the available memory and the service could not be started.

**Table 3:** Used memory after a restart of E-PIX of version 2.8.2 for various numbers of patients

Number of registered patients (million)	Used memory (in GiB)
10	27.59
15	48.81
20	78.72
25	82.18
30	110.83

The relation is not linear and depends on the remaining available memory

### Processor

The processor utilization was nearly constant, independently of the number of registered patients. All processor cores were used. Thus, E-PIX could parallelize the workload. The average processor utilization by E-PIX was in all three benchmarks between approximately 18 percent and 22 percent. Thus, the processor was not fully utilized, so that processor performance was not a limiting component for the whole process. Hence, a processor with lower performance could achieve comparable runtimes, when it is utilized to a larger percentage. In addition, it could be determined that other processes had no influence on the whole process. The sum of idle process and E-PIX process were nearly 100 percent, thus even the database had low influence on the processor workload.

## Discussion

With E-PIX, several million patients can be managed on an appropriately powerful system. The performance of the processor is not a limiting component, but the capacity of the memory limits the processing of very large databases.

The three benchmark scenarios showed the behavior of the system, when patients were registered sequentially without restarts, which is the expected use case. The cyclic restarts of E-PIX showed the behavior in cases of a necessary restart. The last benchmark with pre-filled databases shows how many patients could be managed with E-PIX. However, this benchmark showed only the behavior of registering 100.000 patients after a restart. A benchmark without restarts and pre-filled database could determine the runtimes and memory usage of this entire process, but would need much more time.

No data in terms of performance and number of manageable patients were available for E-PIX before. These data were collected and allow for an assessment of the suitability of E-PIX especially for large research projects. For even larger databases, the memory must be increased.

The sequential registration of patients is not the typical use case. Studies with larger sample sizes are usually multicenter, and therefore multiple sites register patients simultaneously in a productive environment. Hence, our measurements represent the optimal use of the E-PIX system. However, we also performed tests with parallel requests. These requests were proceeded in two ways: First, multiple PII were sent and registered to E-PIX in one request. Here, a bug was found, which prevented the transmission of multiple patient data to E-PIX in one request that included the address data of the patients. This bug was communicated to the developers of the E-PIX and has been fixed. Afterwards multiple PII could be transmitted to E-PIX, which led to lower runtimes, due to the saved overhead of less connections. Second, multiple requests were sent simultaneously to E-PIX. This represents the typical use case in a productive environment. It was found that requests were processed in parallel by E-PIX, but the record linkage process is not designed for multiple requests. The process of record linkage is multithreaded, but only within one registration, not over multiple requests with several registrations. Since in theory, any new patient entry can cause a conflict with any other, each request has to wait for a complete record linkage of all previous requests. This led to similar runtimes in terms of the processing time, but to higher waiting times. However, this process could be principally executed in parallel, thus this circumstance was communicated to the developers, too. The resulting optimizations of E-PIX could not be considered in this paper, but are intended to reduce the runtimes and lead to better utilization of processor cores. In the first step, all identities of a request are compared with the previously received identities of previous requests that have not yet been fully processed. If there is at least one possible match, then the requests are processed sequentially. If no match is determined, then the request is processed parallel.

The presented measurements are based on the E-PIX version 2.8.2, the current version 2.9 of E-PIX includes the mentioned optimizations.

The basis of the used data for PII were anonymized real patient data from the Charité. These data could includes faults, but we did not add artificial errors. The only difference to real patient data is that patients with missing values were excluded. The reason was that patients with missing values in PII would not have eligible for record linkage and, consequently would not have been registered. The exclusion of missing values created a somewhat too optimistic result with respect to performance, but would have no impact on the maximum size of database. In addition, we did not simple add duplicates, because these would only have led to runtimes due to record linkage, but would not have increased the size of database and therefore would have had no impact on further registrations. However, the aim of the benchmark was to determine the maximum possible database size.

We screened the international scientific literature for any examples of runtime measurement and maximum database size analyses for identity management systems—but were not successful.

The disk utilization was not considered in the benchmark scenarios. Even if most data will be cached or held in memory, the disk utility should be considered for further optimizations. The test system used hard drive disks. Solid drive disks could lead to reduced runtimes.

## Conclusions

The goal to register at least 20 million patients into E-PIX was accomplished. In addition, the E-PIX provides sufficient capacity for further extension. Even if the database contains 30 million patients, it is possible to register more than 16 thousand new patients per day. E-PIX is a sufficient solution to handle the expected number of patient registrations in the future. The application of E-PIX is intended for the research context. A central identity management does not require the described test hardware to store the patients already registered. In particular, it is not necessary to use such a powerful processor layout, because in the tested configuration processor capacity was underutilized. In benchmark 3, the application server could not be started due to a lack of memory necessary to handle the large number of 35 million registered patients. A computer system with more memory could solve this issue. In addition, the current upgrade from E-PIX (version 2.9) mitigates this problem by reducing memory usage. In summary, the benchmarks showed that it is possible to manage multi-million patients in large research projects at the Charité with E-PIX. Moreover, the E-PIX could handle large databases with high performance, it can be applied to central identity management tasks both within and between institutions, including large biobanks, prospective clinical patient cohorts, blood and bone marrow donor registries, etc.

## Data Availability

The E-PIX^®^ is licensed under AGPLv3 and open source. The code is available at https://github.com/mosaic-hgw/E-PIX. A runnable E-PIX as a Docker container is available in the TMF ToolPool Gesundheitsforschung at https://www.toolpool-gesundheitsforschung.de/produkte/e-pix.

## Abbreviations

ZeBanC: Central Biomaterial Bank of the Charité
E-PIX: Enterprise Identifier Cross-Referencing
PII: Personally identifying information
MPI: Master Patient Index
JVM: Java Virtual Machine
WPR: Windows Performance Recorder
ETW: Event Tracing for Windows
